# Efficacy and safety of ondansetron for morning sickness in pregnancy: a systematic review of clinical trials

**DOI:** 10.3389/fphar.2023.1291235

**Published:** 2023-10-23

**Authors:** Ahmed M. Ashour

**Affiliations:** Pharmacology and Toxicology Department, College of Pharmacy, Umm Al-Qura University, Makkah, Saudi Arabia

**Keywords:** ondansetron, pregnancy, clinical trials, serotonin, vomiting, morning sickness

## Abstract

**Background:** Ondansetron is a selective antagonist of the serotonin 5-HT3 receptor that is commonly used to treat morning sickness. It is estimated that 70%–80% of pregnant women suffer from morning sickness, a condition characterized by nausea and vomiting. However, it is still controversial regarding its safety during pregnancy, and continued research will be necessary to fully understand the risks and benefits associated with its use. Therefore, we aimed to identify and provide details of the efficacy and safety of ondansetron in clinical trials.

**Methods:** A search was conducted of the ClinicalTrials.gov database on 13 April 2023, using the search term “ondansetron and pregnancy.” Inclusion and exclusion criteria were defined to identify relevant clinical trials. The inclusion criteria encompassed clinical trials related to pregnancy that utilized ondansetron as a treatment, while other clinical trials were excluded from consideration. All data extractions such as study title, study status, study type, intervention details, and outcome were collected.

**Results:** A total of 18 clinical trials were identified, of which only 6 focused on studying the effects of ondansetron. Their respective study titles, statuses, conditions, interventions, outcome measures, and enrollment sizes have been written in detail. The information collected from these trials will contribute to our understanding of the potential benefits and risks of ondansetron in the context of pregnancy and its complications.

**Conclusion:** Ondansetron has been shown to be an effective treatment for nausea and vomiting, including pregnancy-related morning sickness. Further research is needed to better understand the potential risks and benefits associated with its use in pregnant women.

**Systematic Review Registration:** ClinicalTrials.gov, identifier

## Introduction

Ondansetron is a selective serotonin receptor antagonist that prevents nausea and vomiting associated with chemotherapy, radiotherapy, and surgery and is commonly used to treat morning sickness (Wolf, 2000). It is estimated that 70%–80% of pregnant women suffer from morning sickness, a condition characterized by nausea and vomiting (Lee and Saha, 2011). Conditions such as malnutrition and dehydration can cause risks to the health of both the fetus and the mother (Maltepe, 2014). The hormonal changes that occur during pregnancy, such as elevated levels of chorionic gonadotropin (hCG), may result in increased levels of serotonin in the body, which, in turn, might be involved in causing maternal nausea and vomiting during pregnancy (Cengiz et al., 2015; Thibeault et al., 2019). Ondansetron works in the brain by selectively binding to specific serotonin (5-HT3) receptors (Simino et al., 2016). These are located on the terminals of the vagus nerve, which innervates the gastrointestinal tract (Griddine and Bush, 2022). Therefore, the serotonin receptors are blocked, inhibiting serotonin release. Serotonin, also known as 5-hydroxytryptamine (5-HT), is a neurotransmitter that regulates nausea and vomiting (Griddine and Bush, 2022). By preventing serotonin from binding to its receptors, ondansetron reduces nausea and vomiting (Yokoi et al., 2017). Even though the FDA initially approved ondansetron only for treating chemotherapy- and surgery-related nausea and vomiting (Hesketh et al., 2017), off-label prescribing of the drug for morning sickness has occurred in some cases (Griddine and Bush, 2022).

Ondansetron is, however, still controversial regarding its safety during pregnancy, and continued research is necessary to fully understand the risks and benefits associated with its use (Parker et al., 2018). Several reports have used different studies to determine ondansetron’s effectiveness in treating morning sickness in pregnant women (Colvin et al., 2013; Kennedy, 2016). The results of these studies have been promising, showing that ondansetron is an effective treatment for morning sickness. It is well tolerated, with few side effects, and can be taken in pill or injection form (Slattery et al., 2022). Even though ondansetron appears to be effective, safety concerns have been raised regarding its use during pregnancy (Michie and Hodson, 2020). Studies have found that it may increase the risk of cardiac arrhythmias and congenital disorders if prescribed in the first trimester of pregnancy (Kaplan et al., 2019). Some theories have been proposed that long-term exposure to ondansetron and continuous inhibition of serotonin can affect some physiological processes, including fetal development. However, the overall risk appears to be low, and other studies have reported conflicting results (Danielsson et al., 2014; Freedman et al., 2014).

It is important to remember that despite ondansetron’s apparent effectiveness at reducing nausea and vomiting caused by morning sickness during pregnancy, its safety is still under debate. For this reason, the severity of a patient’s symptoms and alternative treatment options should be considered before prescribing ondansetron during pregnancy (Ernst, 2019; Solihah et al., 2023). Regular monitoring of the patient’s condition is recommended to ensure the safety of the mother and baby. Finally, healthcare providers should provide support to the patient throughout the duration of the treatment. Using clinical trial results from ClinicalTrials.gov, this systematic review examines the efficacy and safety of ondansetron for morning sickness during pregnancy.

## Methods

### Search strategy

A comprehensive investigation was carried out on ClinicalTrials.gov on 13 April 2023, to identify all pertinent studies about the utilization of ondansetron (commonly known as Zofran) as a therapeutic intervention for ailments or conditions associated with pregnancy. The search was conducted by inputting the term “pregnancy and ondansetron” into the search engine of the website to yield relevant results.

### Systematic review search results

The process of identifying pertinent clinical trials relied on the specific criteria outlined in the research area of interest. The inclusion criteria encompassed clinical trials related to pregnancy that utilized ondansetron as a treatment, while other clinical trials were excluded from consideration.

### Data extraction

Data such as study title, study status, study type, intervention details, and outcome.

## Results

### Analysis of the number of relevant clinical trials

As of 13 April 2023, a comprehensive search on ClinicalTrials.gov revealed 18 registered clinical trials specifically related to pregnancy and its complications. These trials focused on ondansetron as a potential treatment and were carefully identified and documented for further analysis. Among them, only 6 trials were dedicated to studying the effects of ondansetron, as highlighted in Table 1, thus aligning with our research objectives and meeting our study inclusion criteria. It is noteworthy that all 6 studies have been reported as completed clinical trials. Their respective study titles, statuses, conditions, interventions, outcome measures, and enrollment sizes have been detailed in Table 1.

**TABLE 1:** Data from https://clinicaltrials.gov, updated on 13 April 2023.

#	Title	Status	Conditions	Interventions and dosing	Outcome measures	Study phase	Number of participants	Gestational weeks	Year
1	“Ondansetron VS. Doxylamine and Pyridoxine in Treating Nausea of Pregnancy” (The National Library of Medicine, 2013a)	Completed	Pregnancy with vomiting	*Ondansetron	Reduction of nausea on the VAS (Visual Analog Scale)	Not applicable	36	Less than 16 weeks pregnant by last menstrual period or ultrasound	2013
				*Placebo (The dose is 4 mg ondansetron and a placebo capsule orally 3 times daily (TID) for 5 days)	Reduction in nausea and vomiting				
2	“Study of Drug Concentration of Ondansetron and Cefazolin in Mothers and Neonates” (The National Library of Medicine, 2016)	Completed	Pregnancy Complications	Phlebotomy (Post-delivery blood samples will be taken from the mother, umbilical artery and vein, and neonate along with other clinical labs)	Pharmacokinetics (PK) results for cefazolin and ondansetron in maternal blood specimens	Not applicable	20	Term pregnancy (37–42 weeks)	2016
					Identification of placental transfer of studied meds (cefazolin and ondansetron)				
					PK results of neonatal blood specimens				
3	“Validating the Effect of Ondansetron and Mirtazapine in Treating Hyperemesis Gravidarum” (The National Library of Medicine, 2022)	Terminated with an outcome for ondansetron	Hyperemesis gravidarum	Mirtazapine	A change in short-term vomiting and nausea from baseline to day 2 in the group treated with mirtazapine *versus* the placebo group	Phase 2	58	Pregnant women with gestational age between 5 + 0 and 19 + 6 weeks	2022
			Nausea gravidarum	Ondansetron	Change in short term vomiting and nausea from baseline to day 2 () in the group treated with ondansetron *versus* the placebo group				
			Vomiting during pregnancy	Placebo	Change in nausea and vomiting from baseline to Day 14 (+/-1) (long term) in the mirtazapine group *versus* the placebo group				
				Ondansetron dose is 8 mg oral tablet twice a day (BID) for 7 days					
4	“Intravenous Ondansetron to Attenuate the Hypotensive, Bradycardic Response to Spinal Anesthesia in Healthy Parturients” (The National Library of Medicine, 2011)	Completed	Hypotension pregnancy	Ondansetron	Number of participants who experienced adverse events as a measure of “safety and tolerability” dose of vasopressors administered	Phase 3	68	ASA 2—Patients with mild systemic disease	2011
				Placebo	Number of episodes of nausea				
				Ondansetron 8 mg IV once daily (OD)					
				Placebo will be administered prior to placement of the spinal anesthetic					
5	“How Pregnant Women and Their Babies Metabolize Ondansetron Compared to a Group of Non-pregnant Women” (The National Library of Medicine, 2013b)	Completed	Postoperative nausea	Ondansetron dose is 8 mg IV once if needed	Volume of distribution	Phase 2	100		2013
					Estimated pharmacokinetic parameter				
					Metabolic clearance of ondansetron				
6	“Can Ondansetron Prevent Neonatal Abstinence Syndrome (NAS) in Babies Born to Narcotic-dependent Women” (The National Library of Medicine, 2020)	Completed	Narcotic addiction	Ondansetron	Number of participants with neonatal abstinence syndrome	Phase 2	196	Neonate gestation age between 37 weeks and 41 weeks and 6 days at birth	2020
			Neonatal abstinence syndrome (NAS)	Placebo	Length of hospital stay				
				If pregnant women have not delivered within 4 h of receiving the IV study medication, they may be given a second dose of IV ondansetron	Total dose of narcotic needed to treat NAS symptoms				
				Out of the 90 mother/baby pairs, 50% will receive ondansetron; the study medication will always be the same for mother and baby	Number of participants requiring adjunctive medication to treat NOWS				

### Participants

A total of 508 adult female participants from a search of ClinicalTrials.gov were included in the systematic review.

### Interventions

Along with ondansetron, some studies have used mirtazapine and metoclopramide for different purposes.

## Discussion

In recent years, ondansetron has received increasing attention for its efficacy and safety in treating morning sickness in pregnancy (Kennedy, 2016). Approximately 80% of pregnant women experience morning sickness, or nausea and vomiting, during pregnancy, particularly during the first trimester (Koren, 2014). Such symptoms often negatively impact their quality of life (Clark et al., 2013). There has been significant interest in ondansetron as several studies over the past few years have shown it to be an effective treatment for morning sickness (Quinlan and Hill, 2003; Kennedy, 2016; Fejzo et al., 2019). Various studies have shown that ondansetron works by blocking serotonin receptors in the brain, alleviating nausea and vomiting symptoms in pregnant women (Heckroth et al., 2021). However, despite the evidence suggesting its efficacy, there is still some concern about its use during pregnancy and it is important to conduct further research to better understand the potential risks and benefits associated with its use in pregnant women (Carstairs, 2016; Kaplan et al., 2019).

Ondansetron’s pharmacokinetics are characterized by rapid absorption, extensive distribution, and hepatic metabolism (Simpson and Hicks, 1996). The drug exhibits swift absorption following oral administration, resulting in peak plasma concentrations manifesting within a span of 1–2 h. Oral ondansetron bioavailability is approximately 60% due to first-pass metabolism in the liver. The drug is extensively distributed throughout the body, with a volume of distribution (Vd) of approximately 140 L. Ondansetron is highly protein-bound, with more than 70% of the drug bound to plasma proteins. The drug is metabolized in the liver by several cytochrome P450 enzymes, primarily CYP3A4 and CYP2D6, and eliminated mainly via feces (Roila and Del Favero, 1995; Christofaki and Papaioannou, 2014).

Ondansetron’s pharmacodynamics are dose-dependent and exhibit a ceiling effect. The drug has a half-life of approximately 4 h and a duration of action of 8–12 h (Lozano, 2013). Ondansetron’s therapeutic dose range is 4–8 mg, and higher doses do not provide additional benefits (Meiri et al., 2007). The drug is generally well-tolerated; the most common adverse effects are headaches, constipation, and diarrhea. Ondansetron may also prolong the QT interval and increase the risk of cardiac arrhythmias (Charbit et al., 2005; Freedman et al., 2014).

## Conclusion

Ondansetron has been shown to be an effective treatment for nausea and vomiting, including pregnancy-related morning sickness. The drug works by blocking serotonin receptors in the brain, reducing vomiting reflex activation. Despite evidence suggesting its efficacy, there are still concerns about its use during pregnancy, particularly during the first trimester. This is due to potential risks to fetal development, including congenital malformations. While some studies have reported a statistically significant association between ondansetron exposure and an increased risk of cardiac malformations, conflicting evidence has also been reported. Further research is warranted to assess the potential risks and benefits associated with the use of ondansetron in pregnant women to ensure that it can be used safely with minimal risk to both the mother and unborn baby.

## Data Availability

The original contributions presented in the study are included in the article/supplementary material, further inquiries can be directed to the corresponding author.
